# Modification of Rhizosphere Microbial Communities: A Possible Mechanism of Plant Growth Promoting Rhizobacteria Enhancing Plant Growth and Fitness

**DOI:** 10.3389/fpls.2022.920813

**Published:** 2022-05-26

**Authors:** Zhaoyu Kong, Hongguang Liu

**Affiliations:** ^1^School of Life Science, Key Laboratory of Poyang Lake Environment and Resource Utilization, Ministry of Education, Nanchang University, Nanchang, China; ^2^Jiangxi Provincial Key Laboratory of Soil Erosion and Prevention, Jiangxi Academy of Water Science and Engineering, Nanchang, China

**Keywords:** PGPR, rhizosphere microbiome, functional diversity, root exudates, chemical diversity, plant–microbe interactions

## Abstract

Plant beneficial bacteria, defined as plant growth-promoting rhizobacteria (PGPR), play a crucial role in plants’ growth, stress tolerance and disease prevention. In association with the rhizosphere of plants, PGPR facilitate plant growth and development either directly or indirectly through multiple mechanisms, including increasing available mineral nutrients, moderating phytohormone levels and acting as biocontrol agents of phytopathogens. It is generally accepted that the effectiveness of PGPR inoculants is associated with their ability to colonize, survive and persist, as well as the complex network of interactions in the rhizosphere. Despite the promising plant growth promotion results commonly reported and mostly attributed to phytohormones or other organic compounds produced by PGPR inoculants, little information is available on the potential mechanisms underlying such positive effects *via* modifying rhizosphere microbial community and soil functionality. In this review, we overviewed the effects of PGPR inoculants on rhizosphere microbial ecology and soil function, hypothesizing that PGPR may indirectly promote plant growth and health *via* modifying the composition and functioning of rhizosphere microbial community, and highlighting the further directions for investigating the role of PGPR in rhizosphere from an ecological perspective.

## Introduction

Plants depend upon the beneficial interactions between roots and microorganisms for nutrient acquisition, growth promotion and disease control under often rapidly changing environments. Plant roots in natural environments are in constant and complex interactions with diverse microbes that inhabit in their vicinity (the soil layers of 0.5–4 mm immediately surrounding the roots), known as the rhizosphere (Lundberg et al., 2012; Kuzyakov and Razavi, 2019). The rhizosphere is one of the most complex ecosystems on earth, considered as a hotspot of plant-microbe interactions. The plant and rhizosphere microbiome have co-evolved for mutual benefits (Eichmann et al., 2021). Plants feed the rhizosphere microbiome with carbon and nitrogen metabolites through root exudation. In turn, beneficial microbes contribute to the nutrient uptake, phytohormone regulation, and biotic and abiotic stress resistance of plant.

**Figure 1:**
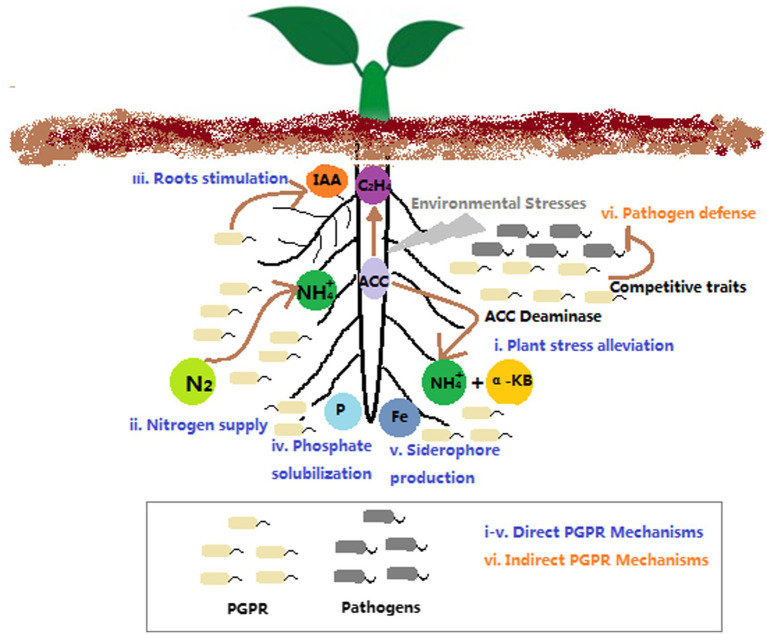
The mechanisms of PGPR improving plant growth and fitness.

Rhizosphere-inhabiting bacteria that have the ability to facilitate plant growth and health are collectively defined as plant growth promoting rhizobacteria (PGPR). PGPR include the rhizospheric bacteria that are free-living (e.g., *Pseudomonas* spp., *Bacillus* spp., *Streptomyces* spp., *Burkholderia* spp., *Azospirillum* spp., etc.), and the bacteria that form specific symbiotic relationships with plants (e.g., *Rhizobium* spp. and *Frankia* spp.; Glick, 2012). PGPR can directly improve plant growth and development *via* increasing available mineral nutrients (e.g., N, P, and Fe) or modulating phytohormone levels, such as auxin, ethylene, cytokinin, abscisic acid, gibberellic acid, etc. (Figure 1). In addition, PGPR can indirectly facilitate plant growth and fitness through their suppressive activity against phytopathogens (Glick, 2012; Kong and Glick, 2017). Typically, the beneficial effects of PGPR on plant growth and health are more pronounced when plants were grown in poor and/or stressed soils (Kong and Glick, 2017). Thus, PGPRs have been extensively studied for their beneficial traits and the potential use in bioaugmentation, biostimulation or biocontrol as microbial inoculums. In particular, PGPR inoculation has been considered as an important strategy for sustainable agriculture, as the successful use of this practice enables to reduce or even eliminate the use of pesticides and/or fertilizers without yield loss (Ambrosini et al., 2016).

It is generally accepted that the effectiveness of PGPR inoculants is associated with their ability to colonize, survive and persist, as well as the interactions with native microbial community in the rhizosphere. Thus, increasing attention has been paid to how PGPR inoculation affects the indigenous microbial community and activity within either rhizospheric soil or bulk soil (Table 1). Since PGPR inoculation can significantly affect root development and exudation (Vacheron et al., 2013; Ray et al., 2018; Alzate et al., 2020; He et al., 2022), it can be expected that PGPR inoculants would modify the community composition of rhizosphere microbiome. Moreover, it is known that the total number of microorganisms can be up to 10^8^–10^12^ per gram of soil (Bloem et al., 1995). A relatively high concentration of PGPR (10^6^–10^12^ CFU/kg soil or 10^6^–10^8^ CFU per seedling or seed) inoculants is always introduced to achieve its effectiveness (Table 1), which can induce changes to rhizosphere characteristics within a certain period (Ambrosini et al., 2016). Furthermore, plant roots can produce a wide range of metabolites which play an important role in shaping the rhizosphere microbiome (Jacoby et al., 2020a). On the other hand, root metabolism and exudation can also change according to the rhizosphere microbiome structure and assembly (Korenblum et al., 2020); even small changes in the microbial community structure might result in large alterations of host phenotypes (Brinker et al., 2019). However, there is still limited information concerning how inoculated PGPR affect rhizosphere microbiome or how subsequent changes in rhizosphere microbiome contribute to improving plant growth and fitness.

**Table 1:** The effects of PGPR inoculants on soil microbial community composition and activity.

PGPR inoculants	Isolation sources	PGP traits	Plants	Inoculant dose	Stress	Plant growth duration	Soil type	Results	Refs
*Neorhizobium huautlense* T1-17	Heavy metal-contaminated soil	IAA, siderophore, ACC deaminase	Chinese cabbages and radishes	5*10^12^ CFU/kg soil	Cd, Pb	2 months	R	Significantly increased the ratio of IAA-producing bacteria	Wang et al. (2016) (G)
*Pseudomonas* sp. SUT 19; *Brevibacillus* sp. SUT 47	Roots of forage corn	IAA, N_2_-fixing, ACC deaminase, P solubilization only for SUT19	Forage corn	10^8^ CFU per seedling/seed	/	1–2 months	R	No influence on microbial community structure	Piromyou et al. (2011) (L and F)
*Pseudomonas* sp. S2-3 and UW4; *Burkholderia* sp. S6-1	Farmland polluted by acidic mine drainage	IAA, P solubilization, Ammonia production, ACC deaminase for S6-1 and UW4, siderophore only for S6-1	*Brassica juncea*	3.75*10^9^ CFU/kg soil	Cu, Pb, and Zn	1–100 days	B	Significantly changed the bacterial community composition 1 day after inoculation, with minor changes continuing to be observed 10 days after inoculation; increased the complexity and stability of co-occurrence network	Kong et al. (2019) (L)
*Burkholderia phytofirmans* PsJN	Onion roots	ACC deaminase, IAA	Maize	Seeds incubated in 10^9^ CFU/ml for 90 min	Cd, Pb and Zn	69 days	R	Affected rhizosphere microbiome diversity only to a minor extent	Touceda-González et al. (2015) (G)
Mix culture of *Bacillus aryabhattai* and *Bacillus megaterium*	Culture collection	P solubilization	*B. juncea*	1.67*10^9^ CFU/ kg soil	Cd	1–8 weeks	R	Significantly changed the species diversity and richness indices of microbial community	Jeong et al. (2013) (L)
*Azospirillum brasilense* Sp6	Katholieke Universiteit Leuven, Belgium	IAA	Quailbush	1.2*10^6^ CFU/seed	Metals	15, 30 and 60 days	R	Induced a significant change in the DGGE profiles of rhizosphere microbial community	De-Bashan et al. (2010) (G)
*Sinorhizobium meliloti* 4H41 and/or *Rhizobium gallicum* 8a3	Common bean nodules	N_2_-fixing	Common bean	10^8^ CFU/plant	/	0, 1 and 2 months after _inoculation	B	Significantly affected the composition of the bacterial and *Rhizobiaceae* communities	Trabelsi et al. (2011) (F)
*Clavibacter* sp. MTR-21A, *Rhodanobacter* sp. MTR-45B, and *Arthrobacter* sp. K4-10C and MTR-44	Rhizosphere of quailbush plants	IAA and siderophores for all strains; P-solubilization for MTR-21A and MTR-44; ACC deaminase only for MTR-44	Quailbush and buffalo grass	2*10^7^ CFU/seed for alginate-encapsulation	Metals	75 days	R	Significantly influenced the development of the rhizosphere community structure	Grandlic et al. (2009) (G)
*Azospirillum lipoferum* CRT1	Commercial inoculants	/	Maize	3*10^7^ CFU/seed	/	7, 35 and 65 days	R	Modified the composition of the resident bacterial community of the rhizosphere	Baudoin et al. (2009) (F)
*Paracoccus versutus* NM01 and *Aeromonas caviae* NM04	As-polluted soils	IAA, siderophore, and P solubilization	Fern	/	As	4 weeks	R	Displayed higher bacterial diversity indices (ACE and Chao1)	Marwa et al. (2020) (G)
*Pseudomonas fluorescens* MC46	Rhizosphere of *Vigna unguiculata* subsp. *sesquipedalis*	Ammonia production, P-solubilization, siderophore, IAA, EPS	Mung bean	2 × 10^8^ CFU per pot	Triclocarban	5 weeks	B	Enhanced soil enzyme activities	Sipahutar et al. (2018) (G)
*A. brasilense* 40 M and 42 M	Roots of field-grown maize	/	Rice	6*10^9^ CFU/kg seed	/	35 and 117 days	R	Significantly increased the percentage of microaerophilic diazotrophs; significantly changed the taxonomic structure and the functional diversity of rhizosphere microbial community	de Salamone et al. (2010) (F)
*Enterobacter* 15S; *Pseudomonas* 16S	Horticultural soils	IAA, P solubilization, siderophores production	Tomato	10^6^ CFU per seedling	/	40 days	R	Induced a deterministic effect on the functional diversity of rhizosphere microbiome	Alzate et al. (2020) (G)
*Enterobacter* 15S; *Pseudomonas* 16S	Horticultural soils	IAA, P solubilization, siderophores production	Tomato	10^6^ CFU per seedling	NaCl	40 days	R	Increased the content of ROS-scavenging and antioxidant compounds, and improved the facilitation of Fe acquisition by inoculation of *Pseudomonas* 16S	Alzate et al. (2021) (G)
*A. brasilense*	Maize rhizosphere	IAA, siderophores, ACC deaminase	Maize	10^11^ CFU/kg seed	/	62 and 132 days	R	Increased the number of microaerophilic nitrogen fixing microorganisms; modified the physiology of the rhizosphere microbial communities	Di Salvo et al. (2018a) (F)
*A. brasilense* 40 M and 42 M	Roots of field-grown maize	/	Wheat	5.55*10^9^ CFU/kg seed	/	88 and 133 days	R	Modified both physiology and genetic structure of rhizosphere microbial communities.	Di Salvo et al. (2018b) (F)
*A. brasilense* Az1 and Az2, *Pseudomonas fluorescens* Pf	Commercial inoculants	/	Wheat	5.5–10.5*10^9^CFU /kg seed	/	106, 136, 155 days	R	No influence on culturable actinomycetes and bacteria, but changed the number of culturable fungi and the carbon-source utilization activities of microbial communities	Naiman et al. (2009) (F)
*Acinetobacter pittii* and *Escherichia coli*	Culture collection	P solubilization	*Solanum nigrum* L.	4–5 × 10^10^ CFU/plant	Cd	30 and 60 days	R	Enriched dominant microbial taxa with plant growth promotion function and keystone taxa related to Cd mobilization; up-regulated the expression of genes related to bacterial mobility, amino acid metabolism, and carbon metabolism among rhizobacterial community	He et al. (2022) (G)
*Bacillus subtilis*, *Paenibacillus polymyxa*	Commercial inoculants	/	Wheat	30 kg ha^−1^	High P	At regreening, flowering, and harvest stages	R	Significantly enriched various bacterial genera	Chen et al. (2021) (F)
*Bacillus amyloliquefaciens* FH-1	Rhizosphere of tea tree	N_2_-fixing, inorganic P, K solubilization, siderophore, ACC deaminase	Cucumber	10^8^ CFU/ g soil	Coastal saline-alkali soil	35 days	R	Reduced the rhizosphere bacterial diversity, increased Proteobacteria, and decreased Acidobacteria; increased bacteria-bacteria interactions	Wang et al. (2021) (L)

Soil type: R, rhizospheric soil; B: bulk soil.

Experimental conditions are designated (L) for more controlled laboratory conditions, (G) for greenhouse conditions, or (F) for field trials.

Here, the effects of PGPR inoculation on the microbial properties and functioning of rhizosphere is reviewed and discussed. The effect of PGPR inoculants on the microbial structural and functional diversity and chemical diversity in the rhizosphere are described in detail. Ultimately, understanding the modification effects of PGPR inoculation on rhizosphere microbiome, and their subsequent role in the rhizosphere functioning is key to a deep sight into the plant growth promotion (PGP) mechanisms of PGPR. It is essential for the establishment of strategic plant-microbe partnership improving plant health and fitness under rapidly changing environments.

## Effects of PGPR Inoculation on the Structural Diversity of Rhizosphere Microbial Community

Current studies revealed the great variability concerning the effects of PGPR inoculation on the structural diversity of rhizosphere microbial community due to the distinct PGPR inoculants, plant species and soil conditions (Table 1). It is known that the success of PGPR inoculation highly depends on the specific interactions between host plants and bacteria, which are mediated through root exudates and bacterial competitive traits (Ambrosini et al., 2016). Good colonizers of roots and rhizosphere are expected to have high growth rates and be more efficiently in resource use. They may potentially affect the resident microbial community in the rhizosphere. For example, a filed study on the ecological impact of PGPR inoculation on resident bacteria found that inoculation of maize seeds with PGPR strain *Azospirillum lipoferum* CRT1 caused a significant shift in the ARISA fingerprints of the indigenous rhizobacterial community at 7 and 35 days (Baudoin et al., 2009). Similarly, inoculation with arsenic (As)-tolerant PGPR strains increased the rhizobacterial alpha-diversity indices after 4 weeks and enhanced As phytoextraction effect of *Adiantum cappillus-veneris* plants (Marwa et al., 2020). Significant impacts of phosphate-solubilizing bacterial inoculants on the rhizosphere microbiome of *Brassica juncea* were also observed after inoculation for 8 weeks, suggesting that it takes some time for the inoculated PGRR to survive, and function during phytoextraction of Cd-contaminated soil (Jeong et al., 2013). However, if the inoculants have a low efficiency in resource use, or are unable to survive in highly diverse communities, minor or no changes would happen to the diversity or structure of rhizosphere-related microbial community following PGPR inoculation (Piromyou et al., 2011; Touceda-González et al., 2015). Moreover, some researchers also have concluded that the microbial communities in the rhizosphere are highly buffered against the inoculation of non-native bacteria (Björklöf et al., 2003).

Although inoculation with PGPR affect rhizosphere microbiome only to a minor extent, the plant growth promotion effects of inoculants have also been reported (Piromyou et al., 2011; Touceda-González et al., 2015). Even if the abundance and persistence of the PGPR inoculants over time are not guaranteed, the promotion of plant growth was observed (Chen et al., 2022). This contradiction raises a question of how the long-term plant growth promotion is maintained even after PGPR inoculants disappeared from the rhizosphere? A recent study reported a possible mechanism that PGPR-induced DNA methylation modifications in roots contributing to the long-term plant growth promotion effects, while neither the colonization of inocula nor the changes in rhizosphere microbiome was necessary for the promotion process (Chen et al., 2022). Furthermore, several studies have reported that PGPR inoculants have potential to cause changes in specific subgroups of microbial community in the rhizosphere (Naiman et al., 2009; de Salamone et al., 2010; Wang et al., 2016; Di Salvo et al., 2018a). For instance, the inoculation with PGPR strain *Neorhizobium huautlense* T1-17, which possess multiple PGP traits such as IAA, siderophore and 1-aminocyclopropane-1-carboxylate (ACC) deaminase activity, significantly increased the proportion of IAA-producing bacteria in the rhizosphere of Chinese cabbages and radishes (Wang et al., 2016). Similarly, a significant increase in the number of microaerophilic nitrogen fixing microorganisms was observed in the rhizosphere of PGPR-inoculated crops, such as maize (Di Salvo et al., 2018a) and rice (de Salamone et al., 2010). In addition, PGPR inoculation has no influence on culturable actinomycetes and bacteria, but changed the number of culturable fungi in the rhizosphere of wheat plants (Naiman et al., 2009). Moreover, our pervious study on phytoremediation of heavy metal-contaminated soils suggested that PGPR inoculation greatly changed the bacterial community composition only within 10 days after inoculation, maintaining a microbial community profile (at the phylum level) similar to what was observed in the original soil in the late stage (Kong et al., 2019). However, enhanced co-occurrence associations were observed in the PGPR-inoculated bacterial community network, indicating the bacterial community had more complex and compact associations in the presence of PGPR inoculants during the phytoremediation process. Furthermore, the inoculation of phosphate-solubilizing bacterial strains enriched dominant microbial taxa with PGP function and keystone taxa related to Cd mobilization during the phytoremediation of soil Cd (He et al., 2022). These above findings cannot be comprehensively explained by the competition between the resident and added strains. It reminds us that although addition of specific bacteria may result in competitive pressure on the native microbial community in the case of nutrient limitation, the cooperation between native and inoculated strains also needs to be considered. In fact, an increasing number of cooperative behaviors are being discovered among microorganisms in laboratory (Sieuwerts et al., 2008; Harcombe, 2010; Poltak and Cooper, 2011) or natural microbial communities (Schink, 2002; McCutcheon and Moran, 2008; Morris et al., 2013), which can significantly affect the fitness of interacting partners. For instance, metabolic cross-feeding interactions are ubiquitous in natural microbial communities. PGPR possess beneficial traits for better interaction with plants, which involves producing phytohormones that affect root growth dynamics, chelating compounds that aid in nutrient acquisition, or synthesizing polymeric substances to form biofilms (van Gestel et al., 2014). The by-products of PGPR inoculants are released into the rhizosphere that can be used by other microorganisms as nutrients or energy source. For example, ACC deaminase-producing PGPR can facilitate plant growth and development through conversion of the immediate ethylene precursor ACC into α-ketobutyrate and ammonia, which can be used by other associated microorganisms as substrates (Nascimento et al., 2018). Furthermore, the horizontal transfer and microevolution of ACC deaminase genes not only occur between bacteria, but also may occur between bacteria and fungi (Nascimento et al., 2014). Therefore, establishing symbiotic or synergistic mutually beneficial relationships with the added PGPR strains might be an adaption and survival strategy for some microbes, particularly in face to environmental stresses. Thus, the enhanced cooperative interactions in the PGPR-inoculated soils may contribute to the plant growth-promoting effects of PGPR, particularly in stressful environments.

## Effects of PGPR Inoculation on the Functional Diversity of Rhizosphere Microbial Community

Although PGPR inoculants have previously been examined for their impacts on the structural diversity of rhizosphere microbial community, there is limited information of the effects of the addition of inoculants on the function of the microbial community. Some researchers suggested that the ecological impacts of PGPR inoculation should receive more attention in order to know how the functional diversity is altered (Naiman et al., 2009; de Salamone et al., 2010; Di Salvo et al., 2018a). The community-level physiological profiling (CLPP) using Biolog EcoPlates is an estimation of the potential catabolism of cultivable microorganisms from environmental samples, which has been widely used to analyze the functional diversity of both soil and rhizosphere microbial communities (Salvador et al., 2017; Mhlongo et al., 2018). The carbon sources tested in the Biolog EcoPlates can be commonly found in plant root exudates (Campbell et al., 1997). It is believed that the majority of primary metabolites in root exudates, including organic acid, sugar and amino acids can be utilized by rhizosphere microbial communities. Some authors have concluded that the physiological profiles of rhizosphere microbial communities of various crops can be modified by PGPR inoculation, such as rice (de Salamone et al., 2010); tomato (Alzate et al., 2020, 2021); maize (Di Salvo et al., 2018a); and wheat (Naiman et al., 2009; Di Salvo et al., 2018b). For example, Alzate et al. (2020) found that carbohydrates, carboxylic acids, amino acids and polymers were the main types of substrates that contribute to the functional diversity of the rhizosphere microbiome between PGPR inoculated and non-inoculated treatments. They suggested that PGPR inoculants can alter the rhizosphere functioning by affecting the root exudation profile, thus interfering in the plant–soil feedback and reshaping the rhizosphere microbiome. These authors also observed an increase in the content of ROS-scavenging and antioxidant compounds, and an up-accumulation of the aphytosiderophore avenic acid in the tomato rhizosphere inoculated with PGPR inoculation under saline stress, which are suggested as the mechanisms responsible for higher plant biomass production in salinity (Alzate et al., 2021). Moreover, microbial communities can produce extracellular enzymes to acquire energy and resources from complex biomolecules in soils. Currently, there is a great interest in using extracellular enzyme activities as indicators of microbial function, since they are relatively simple to measure, sensitive to environmental stress and soil disturbance. A pot soil experiment showed that a triclocarban-degrading PGPR strain *Pseudomonas fluorescens* MC46 not only significantly promoted plant growth and health in triclocarban-contaminated soil, but also enhanced soil enzyme activities, indicating their role in improving soil fertility (Sipahutar et al., 2018). Furthermore, the metabolic function of microbial community can also be predicted by mapping sequences to KEGG database using PICRUSt software (Langille et al., 2013). Using this approach, these authors found that inoculated phosphate-solubilizing bacteria up-regulated the expression of genes related to bacterial mobility, amino acid metabolism, and carbon metabolism of the rhizobacterial community during the process of Cd phytoremediation (He et al., 2022). These findings suggested that PGPR inoculation that can modify the metabolic profile of rhizosphere microbiome *via* interfering root exudation patterns, which could be one possible plant growth-promotion mechanism.

## Effects of PGPR Inoculation on the Chemical Diversity in the Rhizosphere

In the rhizosphere, a complex interaction network co-exists among plant roots, soil microbes and the soil. Governed by root exudates, these interactions primarily mediate plant growth, development and fitness. Root exudates mainly consist of low molecular weight organic compounds, which include amino acids, organic acids, sugars, phenolics and an array of secondary metabolites, and high molecular weight compounds such as polysaccharides and proteins (Walker et al., 2003). Plants actively modulate qualitative and quantitative root exudation profiles which can modify the rhizosphere properties to adapt to the environmental conditions (Vives-Peris et al., 2020). In addition to primary metabolites that can be used by rhizosphere microorganisms, secondary metabolites have often been believed to play an active role in shaping the rhizosphere microbiome assembly, such as flavonoids (Hassan and Mathesius, 2012); benzoxazinoids (Hu et al., 2018; Cotton et al., 2019); coumarins (Stringlis et al., 2018; Voges et al., 2018); strigolactones (Nasir et al., 2019); triterpenes (Huang et al., 2019); camalexin (Koprivova et al., 2019); ethylene (Chen et al., 2020), etc. (Jacoby et al., 2020b; Eichmann et al., 2021).

The studies on metabolic variances in plant roots as a driver of root-associated microbiome have developed rapidly in recent years due to the advancement in sensitivity of analytical techniques. The field of metabolomics is now routine for the identification of primary and secondary metabolites of plants taking advantage of metabolic footprinting approaches such as liquid chromatography (LC)–mass spectrometry (MS; LC–MS), gas chromatography-MS (GC–MS), capillary electrophoresis-MS (CE-MS), nuclear magnetic resonance spectroscopy (NMR), Fourier transform-near-infrared (FT-NIR) spectroscopy, MS imaging (MSI), and live single-cell-MS (LSC-MS). (Pang et al., 2021). On the microbial side, defined synthetic communities (SynComs) have been successfully used in studying plant-microbe interactions (Lebeis et al., 2015; Castrillo et al., 2017; Niu et al., 2017; Liu et al., 2019), which has been considered as an excellent tool to predict the plant phenotypes upon microbial inoculation (Herrera Paredes et al., 2018). For example, previous study using SynComs approach revealed that the defense phytohormone salicylic acid modulates colonization of the root microbiome of *Arabidopsis* by specific bacterial taxa (Lebeis et al., 2015). Insights of the metabolic and signaling feedbacks between plants and their associated microorganisms in the rhizosphere provide new management approaches for biofertilization, biocontrol or bioremediation.

It is now widely accepted that plants can recruit beneficial microbes *via* altering their root exudates which may serve as signals in face to adverse environmental conditions. For example, barley plants selectively recruited fluorescent pseudomonads carrying antifungal traits upon pathogen infection, which leads to a reduced impact by pathogen attack on host plants (Dudenhöffer et al., 2016). Similarly, inoculation with a foliar pathogen *Pseudomonas syringae* pv tomato resulted in significant changes in root exudates of *Arabidopsis*, and these changes lead to the recruitment of beneficial rhizosphere communities (Yuan et al., 2018). On the other hand, the metabolic composition of root exudates can be altered systemically and specifically by different bacterial strains (Korenblum et al., 2020). In a tomato split root system, these authors found that systemic exudation of acylsugars (one secondary metabolite) can be triggered by the local root inoculated with *Bacillus subtilis*. This analysis also revealed that both leaf and systemic root metabolomes and transcriptomes changed according to the rhizosphere microbial community structure. Only a few studies so far have addressed the effect of PGPR inoculation on the metabolic profiles of roots exudates or rhizosphere (Table 2). Nevertheless, these limited studies have shown that PGPR may alter the chemical diversity of root exudates and induce the release of specific compounds involved in recruitment of more beneficial microbes. For example, a recent study using untargeted metabolomics approach to investigate chemical profiles of tomato rhizosphere following PGPR inoculation, in which these authors found that a high amount of phenylpropanoid compounds was accumulated in the rhizosphere, including several compounds involved in PGPR colonization and plant growth promotion (Alzate et al., 2020). The metabolic profiles of plant tissues (including roots, stems, and leaves) following PGPRs inoculation were investigated using ultra-high performance LC–MS and their results revealed that PGPR inoculation induced dynamic changes in the metabolomes of plant involving hydroxycinnamates, benzoates, flavonoids, and glycoalkaloids (Mhlongo et al., 2020). Also, Ray et al. (2018) found that PGPR inoculation modified the phenolic profiles of root exudates of okra, which may attract more beneficial rhizospheric microbiota for better resistance to pathogens. These findings demonstrated that PGPR inoculation can lead to plant metabolic reprogramming, which may contribute to their PGP effects. Since root exudates represent an important source for rhizosphere microorganisms, the variations of rhizosphere metabolites in response to PGPR inoculation subsequently alter functional activities of rhizosphere microbial community as described above.

**Table 2:** The effects of PGPR inoculants on root exudates.

PGPR inoculants	PGP traits	Plants	Inoculant dose	Stress	Plant growth duration	Results	Refs
*Enterobacter* 15S; *Pseudomonas* 16S	IAA, P solubilization, siderophores production	Tomato	10^6^ CFU per seedling	/	40 days	Induced a differential accumulation of a high amount of phenylpropanoid compounds	Alzate et al. (2020) (S)
*Pseudomonas* sp.; *Bacillus amyloliquefaciens* FZB42; *Pseudomonas jessenii* RU47	/	Maize	0.4 or 1.2*10^6^ CFU/kg soil	/	43 days	Induced significant changes in secondary pathways of lipid metabolism	Nebbioso et al. (2016) (S)
*Pseudomonas fluorescens* (N04), *Ps. koreensis* (N19), *Paenibacillus alvei* (T22), and *Lysinibacillus sphaericus* (T19)	IAA, siderophores and P-solubilization	Tomato	OD0.5–0.6, 3 ml per pot	/	24, 48 h	Induced dynamic changes in the metabolomes involving in hydroxycinnamates, benzoates, flavonoids, glycoalkaloids, as well as aromatic amino acids	Mhlongo, et al. (2020) (S)
*Acinetobacter pittii* and *Escherichia coli*	P solubilization	*Solanum nigrum* L.	4–5 × 10^10^ CFU/plant	Cd	30 and 60 days	Significantly increased the concentrations of malic, palmitic, L-proline, lactic, L-alanine and γ-aminobutanoic acid	He et al. (2022) (S)
*Alcaligenes faecalis* BHU 12, BHU 16 and BHU M7	IAA, ammonia production, P-solubilization, and production of hydrogen cyanide and proteolytic activity	Okra	The germinated seedlings dipped into 4*10^8^ CFU bacterial suspension	Pathogen infection	14 days	Modified the phenolic profiles of root exudates	Ray et al. (2018) (S)
*Bacillus pumilus* AS 121, *Pseudomonas Mendocina* AS 40, *Arthrobacter* sp. AS 18, *Halomonas* sp. SL 9, and *Nitrinicola lacisaponensis* SL 11	P solubilization and IAA, siderophore, and ammonia production	Wheat	Seeds incubated in 10^8^ CFU/ml for 3–4 h	Salt	15 and 30 days	Increased presence of individual phenolics (gallic, caffeic, syringic, vanillic, ferulic, and cinnamic acids) and flavonoid quercetin in the rhizosphere; Increased the content of IAA in the rhizospheric soil and root exudates	Tiwari et al. (2011) (S)

Experimental conditions are designated (S) for soil culture or (H) for hydroponics.

Nevertheless, the specific PGP characteristics of the inoculated PGPR strains are not being emphasized well when these authors investigate the variations in chemical diversity of living plant roots or rhizosphere imposed by inoculation with distinct PGPR. It is well known that PGPR may facilitate plant growth and fitness using individual or multiple PGP mechanisms and a particular PGPR strain may employ different mechanisms under different environmental conditions (Kong and Glick, 2017). In addition, the PGP traits of PGPRs varies among different species and strains of the same species and are influenced by culture conditions, growth stage and substrate availability. For example, the levels of tryptophan, vitamins, salt, oxygen, pH, temperature, carbon source, nitrogen source and growth stage are all contributing factors in regulation of IAA biosynthesis by microbial isolates (Duca et al., 2014). Current studies have also showed that the effects of bacterial inoculation on metabolic profiles in plant tissues or rhizosphere are strain-specific (Lucini et al., 2018; Alzate et al., 2020; Mhlongo et al., 2020). Therefore, the specific PGP traits of PGPR inoculants cannot be overlooked when analyzing the effects of PGPR inoculation on metabolic activity profile of root exudates or rhizosphere functioning. For example, there are many studies regarding the effects of bacterial ACC deaminase, which is widespread in plant-associated bacteria and extremely important for bacterial PGP abilities (Glick et al., 1998; Nascimento et al., 2014). ACC deaminase-containing PGPR are found to be enriched in the rhizosphere, and are more abundant in stressed soils than in non-stressed soils (Nascimento et al., 2018). These authors reviewed and discussed the role of ethylene and ACC in plant-bacterial interactions and suggested that ACC and ethylene may act as signaling molecules to recruit specific bacteria to reduce the elevated ACC and ethylene levels, alleviating the stress on plants (Nascimento et al., 2018). A study in intercropping agro-ecosystem confirmed that ethylene produced by peanuts alters the rhizosphere microbial composition and re-assembles the microbial co-occurrence network, which provides more available nutrients to peanut roots and support seed production when grown with heterospecific plant neighbors (Chen et al., 2020). In turn, ACC and ethylene-reducing bacteria can protect plants against the inhibitory effects of various environmental stresses (Glick, 2014). Nevertheless, more studies are necessary to understand how ACC deaminase-producing bacteria affect rhizosphere microbiome assembly *via* modulating plant ACC/ethylene levels, which subsequently may feedback on plant phenotypic traits.

## Conclusions and Future Perspectives

PGPR have been considered as the key elements of rhizosphere engineering for their ability to promote plant growth and fitness under abiotic and biotic stress conditions. In the past 2–3 decades, hundreds of PGPR strains have been isolated, characterized and used to promote the growth and development of a variety of different plants under normal and stressful conditions. With a better understanding of how various PGPR contribute to plant growth, scientists have paid more attention to the effects of PGPR inocula on underground soil microbial community. Although an increasing number of studies have concluded that PGPR inoculation affect rhizosphere microbiomes, it remains unclear whether or how subsequent changes in rhizosphere microbiome contribute to improving the growth and stress resistance of host plants. PGPR inocula may directly affect the composition of rhizosphere microbiome, or they may indirectly affect rhizosphere microbiome composition *via* interfering root exudation patterns, which in both ways can alter the functional activity of the rhizosphere mcirobiome and finally facilitate plant growth and fitness. This suggests the need for a deeper understanding of the mechanisms underlying PGPR-induced plant growth promotion in the rhizosphere. In this regard, the three-way interactions among the PGPR inocula, indigenous rhizosphere microbiome and plant roots need to be integratively studied to understand the plant growth-promoting process. Root exudates can provide the first insights into plant-microbe interactions, and the role of exudates for shaping rhizosphere microbiome has been readily confirmed. Nowadays, new approaches have been developed and allow us to get a deeper insight into PGPR-roots-rhizosphere microbiome interactions. For example, metabolomics approach, especially untargeted can provide detailed information about the composition of root exudates and how they are affected by rhizosphere microbes, which allows us to find key compounds modulating plant–microbes interactions. By combining with metabolomics, plant transcriptomics and rhizospheric bacterial community integrative analyses can provide deeper insights into how inoculants promote plant growth and stress resistance. Furthermore, to get a better understanding of mechanisms underlying PGPR-induced plant growth promotion in the rhizosphere, the specific PGP traits of the used PGPR inoculants should also be addressed, since the influence of PGPR inoculation on rhizosphere microbiome is strain-specific. However, the PGPR inoculants used in most studies possess multiple PGP activities and it is difficult to figure out which activity is more important. In the future, more efforts can be taken in uncovering whether and which specialized molecules or metabolites produced by PGPR are involved in the modification of rhizosphere microbiome using wild-type versus PGP mutants. For example, studies using bacterial mutants impaired in ACC deaminase production have demonstrated that the expression of ACC deaminase can readily impact the colonization of other microorganisms present in the rhizosphere, including symbionts (Nascimento et al., 2018). Ultimately, understanding how PGPR modify rhizosphere microbiome and subsequently feedback on plant phenotypic traits will enable the development of rhizosphere engineering strategies using specific PGPR or signals to modify rhizosphere functioning for a given soil and environment.

## Author Contributions

ZK: conceptualization and writing—original draft preparation. ZK and HL: investigation, visualization, and writing—review and editing. All authors contributed to the article and approved the submitted version.

## Funding

This work was supported by the National Natural Science Foundation of China (42067010, and 41807078), the Program of Young Academic leaders of major disciplines in Jiangxi Province (20212BCJ23039), the National Key Research and Development Program of China (grant no. 2019YFC0605005), the Jiangxi Provincial Natural Science Foundation (20192BAB214007), Postdoctoral Projects in Jiangxi Province (2020KY47). and the Cultivation Plan for Reserved Project of National Science and Technology Award (20212AEI91011).

## Conflict of Interest

The authors declare that the research was conducted in the absence of any commercial or financial relationships that could be construed as a potential conflict of interest.

## Publisher’s Note

All claims expressed in this article are solely those of the authors and do not necessarily represent those of their affiliated organizations, or those of the publisher, the editors and the reviewers. Any product that may be evaluated in this article, or claim that may be made by its manufacturer, is not guaranteed or endorsed by the publisher.
